# Upregulation of PI3K/AKT/PTEN pathway is correlated with glucose and glutamine metabolic dysfunction during tamoxifen resistance development in MCF-7 cells

**DOI:** 10.1038/s41598-020-78833-x

**Published:** 2020-12-14

**Authors:** Lama Hamadneh, Rama Abuarqoub, Ala Alhusban, Mohamad Bahader

**Affiliations:** Faculty of Pharmacy, AL-Zaytoonah University of Jordan, Amman, 11733 Jordan

**Keywords:** Cancer, Chemical biology, Biomarkers

## Abstract

Tamoxifen resistance is emerging as a big challenge in endocrine therapy of luminal A breast cancer patients. In this study, we aimed to determine the molecular changes of *PI3K/AKT/PTEN* signaling pathway during tamoxifen-resistance development using gradually increased doses of tamoxifen in one model, while fixing tamoxifen treatment dose at 35 μM for several times in the second model. An upregulation of *AKT/PI3K* genes was noticed at 30 μM tamoxifen concentration in cells treated with a gradual increase of tamoxifen doses. In the second model, significant upregulation of *AKT1* was seen in cells treated with 35 μM tamoxifen for three times. All genes studied showed a significant increase in expression in resistant cells treated with 50 µM and 35 µM six times tamoxifen. These genes’ upregulation was accompanied by *PTEN* and *GSK3 ß* genes’ down-regulation, and it was in correlation to the changes in the metabolic rate of glucose in tamoxifen-resistant models. A significant increase in glucose consumption rate from culture media was observed in tamoxifen resistant cells with the highest consumption rate reported in the first day of culturing. Increased glucose consumption rates were also correlated with *GLUL* significant gene expression and non-significant change in c-MYC gene expression that may lead to increased endogenous glutamine synthesis. As a result, several molecular and metabolic changes precede acquired tamoxifen resistance could be used as resistance biomarkers or targets to reverse tamoxifen resistance.

## Introduction

Breast cancer patients with luminal A molecular subtype are given endocrine therapy as the first-line treatment^1^. Tamoxifen (TAM), a nonsteroidal antiestrogen drug, is the most-prescribed selective estrogen receptor modulator^2^ used to treat luminal A breast cancer patients, and it decreases mortality rate to 31%^3^.

Unfortunately, Tamoxifen resistance remains a classical challenge in breast cancer treatment^4^, with around 30% of those patients exhibit intrinsic or acquired resistance to tamoxifen treatment^5^. Also, more than half of advanced estrogen receptor breast cancer patients are intrinsically resistant to TAM, and some of the patients acquire tamoxifen resistance (TAM-R) during the treatment^3^.

Many studies have proposed several mechanisms of TAM-R. Among these mechanisms, loss or change in the expression of ERα^6^ and variations in signaling pathways such as growth factor receptor and PI3K/AKT pathway have been reported^3,6,7^ while other mechanisms remain unknown^8^.

The role of impaired activation of PI3K/AKT/*PTEN* pathway have been studied in TAM-R models^6,9,10^. *PI3K/AKT/PTEN* pathway is critical in cancer development and progression, with potential prognostic value to identify the high-risk breast cancer recurrence^11,12^. Knocking down *PTEN* gene expression; a negative regulator of *AKT*, results in increase *PI3K* and *AKT* phosphorylation in ER+ breast cancer cell lines, producing hormone-independent growth and TAM-R^3,6^.

On the other hand, glucose and glutamine metabolic changes are critical in cancer cells growth as they are utilized by the cells through distinct pathways^13^. However, their interactive activity is usually mediated by pyruvate^14^; the end product of glycolysis that would enter into tricarboxylic acid cycle (TCA), while glutamine metabolism would generate α-ketoglutarate, an intermediate of TCA. Glutamate-ammonia ligase (GLUL) catalyzes glutamine synthesis by condensing ammonium to glutamate ^15^.

In this study, we tracked the changes in *PI3K/AKT/PTEN* pathway together with *GSK3ß* and *GLUL* gene expression levels during the development of TAM-R MCF-7, and followed by the correlation with the metabolic rate of glucose consumption in tamoxifen-resistant and tamoxifen-sensitive MCF-7 cell lines in order to have better understanding of these changes during the process of TAM-R development.

## Results

### Tamoxifen resistance development

**Figure 1:**
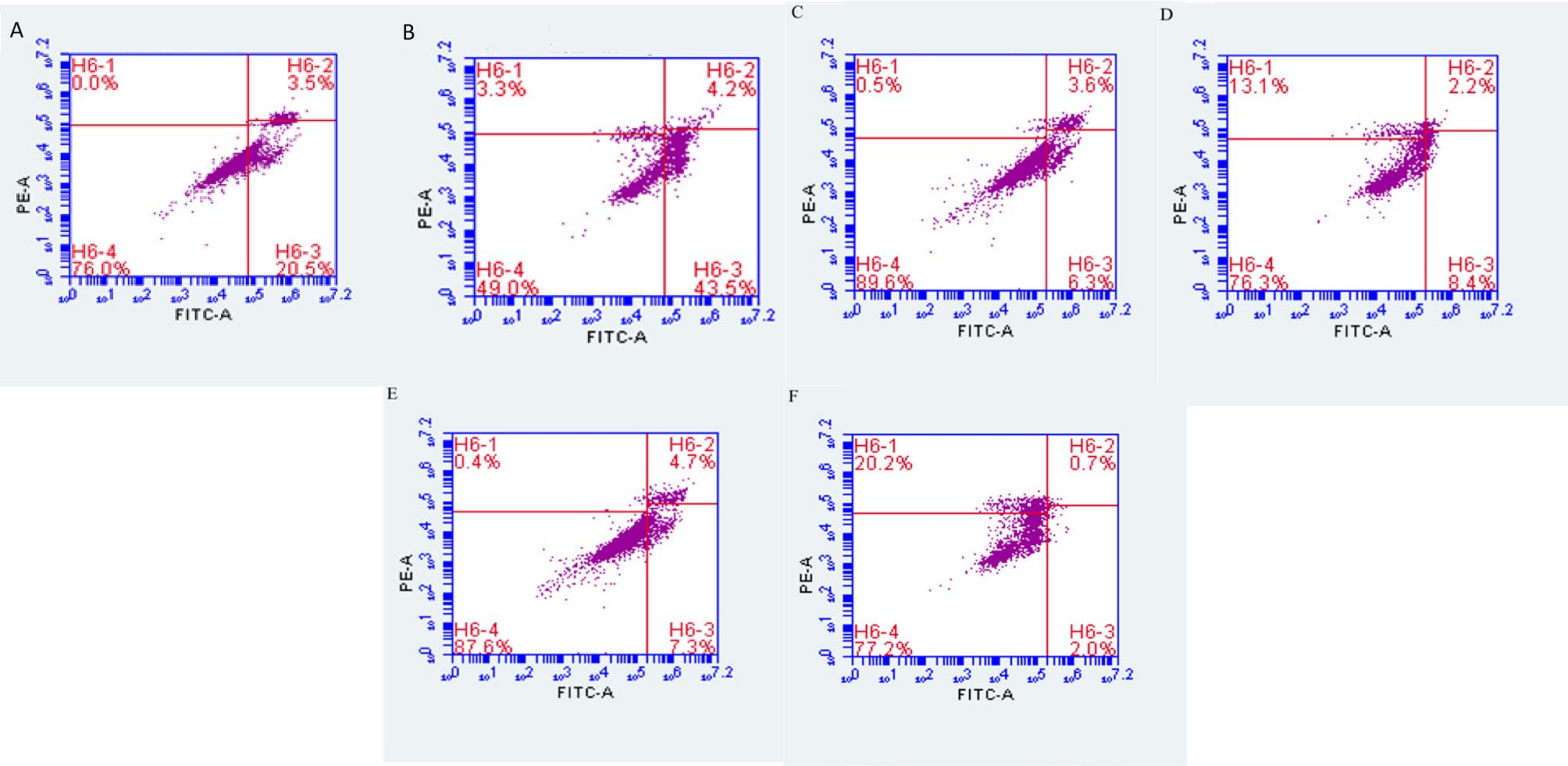
Flow cytometry analysis (**A**) untreated MCF-7 (control) stained with annexin V + PI. (**B**) treated MCF-7 (control)—annexin V + PI. (**C**) untreated TAM-R MCF-7 cells (T50)—annexin V + PI. (**D**) treated TAM-R MCF-7(T50) cells—annexin V + PI. (**E**) untreated TAM-R MCF-7- (T35(6)) cells annexin V + PI. (**F**) treated TAM-R MCF-7(T35(6)) cells—annexin V + PI.

Tamoxifen resistance was produced using two different approaches; the first approach was achieved by a gradual increase of TAM doses starting with 100 nM and reaching the concentration of 50 μM. The second approach was achieved by gradually increasing TAM concentrations up to 35 μM, then fixing the treatment doses at 35 μM of TAM for six times. Resistance was confirmed using flow cytometry, as presented in Fig. 1. Control MCF-7 showed a higher apoptotic response to treatment with TAM with 51% cell death, compared to resistant cells produced by the first and second approaches as the cell death was only 11% and 2%, respectively.

Furthermore, there was a remarkable increase in total necrosis in MCF-7 cells treated with 50 μM and 35(6) μM produced from the first and second approaches as compared to untreated cells; this might be due to the aggressive behavior of these resistant cells through forming multilayers and having higher rates of growth which lead to overgrowth of cells and necrosis.

**Figure 2:**
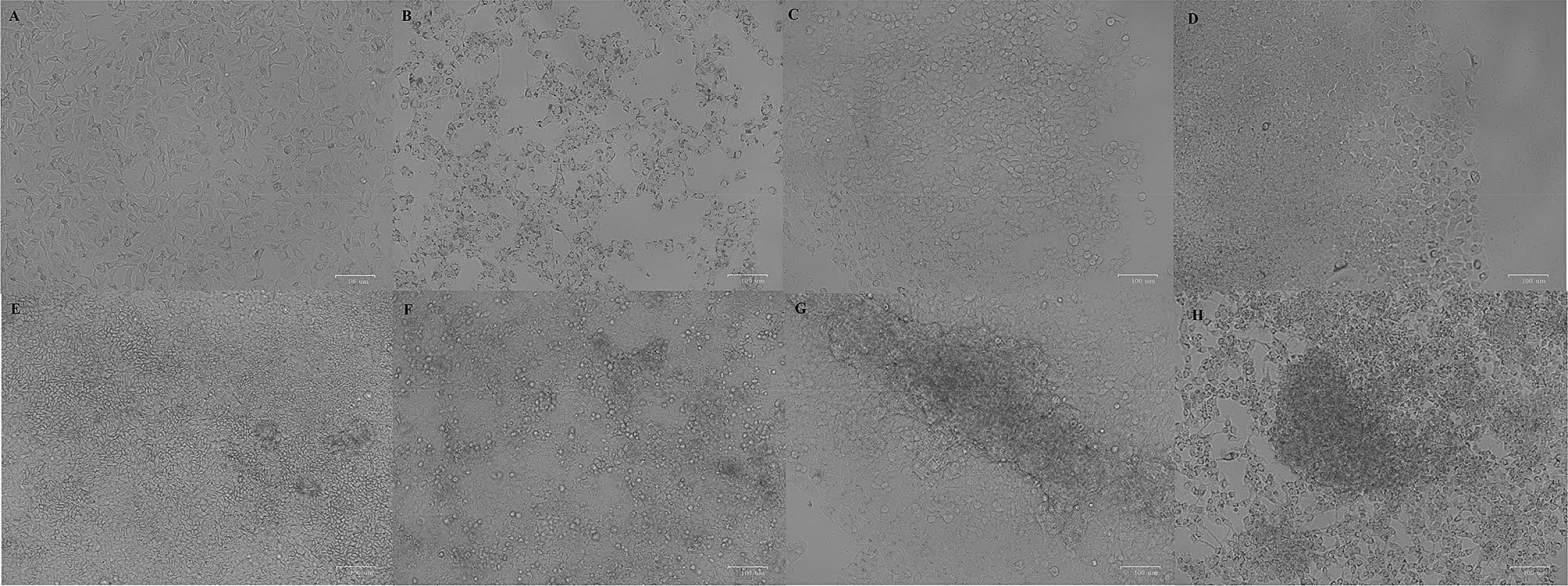
Morphological changes during TAM-R development using two approaches, (**A**) control MCF-7 cells without any tamoxifen treatment. Figures (**B**), (**C**), (**D**) and (**E**) represent MCF-7 cells treated with 30, 40, 45 and 50 μM tamoxifen from the first approach, respectively. MCF-7 cells treated with tamoxifen (**F**) 35 μM two times, (**G**) 35 μM four times and (**H**) 35 μM six times in the second approach. Images were taken using ZOE Fluorescent Cell Imager (Bio-Rad, USA) (Scale bar 100 μm).

Morphological changes in the MCF-7 cell line were observed during the development of TAM-R. Cells' morphology has changed significantly; Cells treated with 30 μM TAM lost their epithelial-like shape and became round. At 40 μM, the cells were aggregated, and the rate of growth was higher than previous treatments. At 45 μM, the cells start to form multilayers. At 50 μM, the cells were more aggressive, with a growth rate much higher than 45 μM. While in cells that were treated with 35 μM TAM for two times (35(2) μM), the cells become more rounded and start to form multilayers. At 35 μM TAM for four times (35(4) μM), the cells were aggregated, and the growth rate was higher than 40 μM. The fastest rate of growth was seen with cells treated with 35 μM TAM for six times (35(6) μM) as shown in Fig. 2. These changes have also been associated with a no effect of higher concentrations of tamoxifen used to treat the cells.

### Gene expression analysis

**Figure 3:**
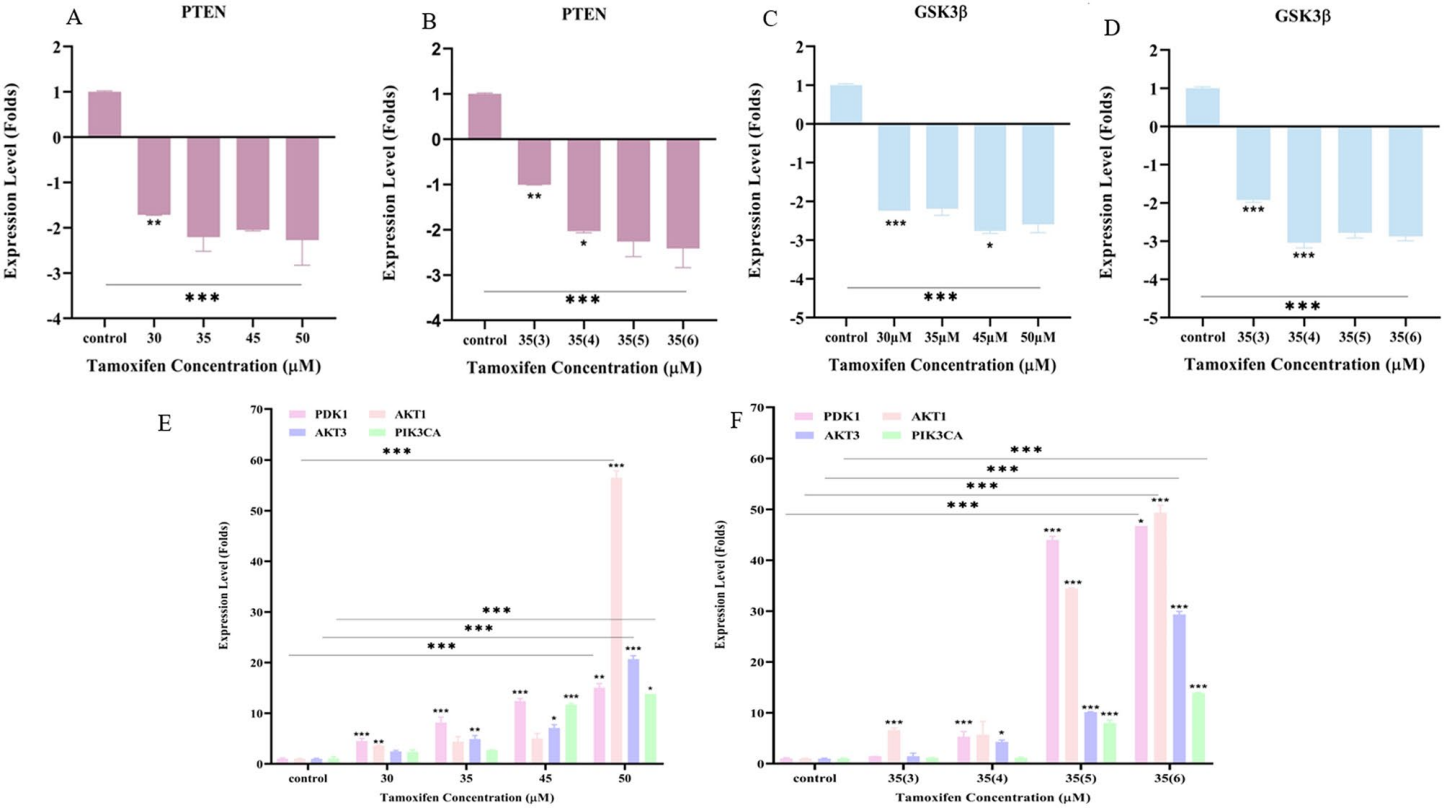
Levels of gene expression (Folds increase and decrease) during the development of TAM-R of *PTEN* (**A**,**B**), *GSK3ß*, (**C**,**D**) and *PDK1, AKT1,AKT3, PIK3CA* (**E**,**F**) using the first and second approaches respectively. Gene expression analysis was performed in triplicates in each run and repeated twice as independent experiments. Folds increase and decrease were presented ± SD. Comparisons were performed between each dose with the previous dose and the last dose with the control untreated sensitive MCF-7 cells via one-way and 2 way annova ANOVA. P* < 0.05, P** < 0.01, and P*** < 0.001.

Gene expression analysis of *PTEN* and *GSK3ß* showed that downregulation started to appear significantly in cells treated with 30 μM and 35(3) μM in the first and second TAM resistance development approaches, respectively. Downregulation of PTEN was accompanied by a significant overexpression of *PDK1* and *AKT1* in the first approach at a concentration of 30 μM, and only *AKT1* at TAM concentration of 35(3) μM in the second approach Fig. 3. Other genes in the* PI3K/AKT/PTEN* pathway started to show significant overexpression in later treatments, as seen in Fig. 3. *AKT3* significant overexpression was seen at a concentration of 35 μM, while PIK3CA significant overexpression was seen at 45 μM TAM concentration in the first approach. In the second approach, *PDK1* and *AKT3* started to show significant overexpression in cells treated with 35(4) μM and PIK3CA was significantly overexpressed in cells treated with 35(4) μM. All genes remained significantly overexpressed in resistant cells produced by both approaches.

**Figure 4:**
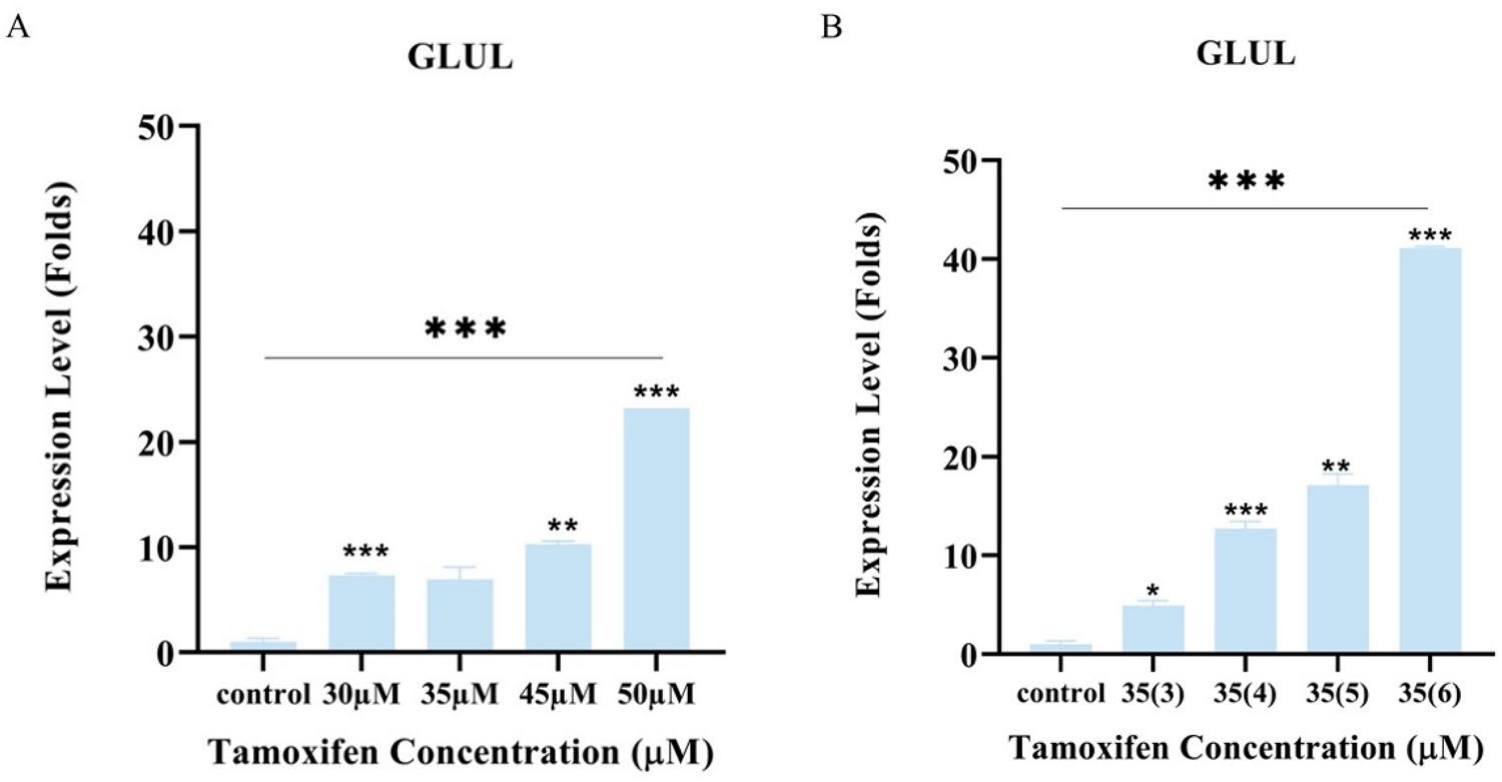
Levels of *GLUL* gene expression (Folds) during development of TAM-R. Gene expression analysis was performed as two independent runs, in each run; samples were analyzed as triplicate and folds increase was presented as ± SD. (**A**) using the first approach of tamoxifen resistance model and (**B**) using the second approach of tamoxifen resistance model. Comparisons were performed between the dose and the previous dose, and the last dose with the control untreated sensitive MCF-7 cells via one-way ANOVA. P* < 0.05, P** < 0.01, and P*** < 0.001.

Similarly, *GLUL* gene expression started to show significant overexpression with concentrations of 30 μM and 35(3) μM in the first and second TAM resistance development approaches respectively, as presented in Fig. 4. On the other hand, *c-MYC* gene expression was obtained from cDNA samples of different tamoxifen treated MCF-7 cells, correlated with *GLUL* gene expression, and they were found to be overexpressed in a non-significant pattern even in tamoxifen resistant cells. These molecular changes together with the morphological and metabolic changes give an insight into the modifications associated with tamoxifen resistance in breast cancer cells.

### Glucose metabolic analysis

**Figure 5:**
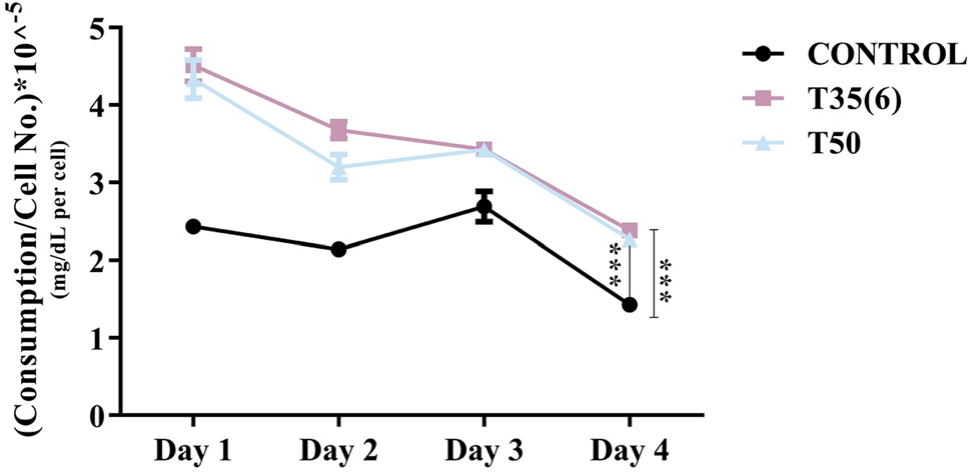
Rate of glucose consumption per day of normalized number of MCF-7 cells. Comparisons were performed between control untreated MCF-7 cells with tamoxifen resistant cells produced from the first (T50) and second approaches T35(6). (P*** < 0.001).

Changes in glucose consumption rate from culture media per cell during four days of culturing conditions were carried out. Glucose consumption rate in TAM-R cells produced using both approaches was significantly higher than the rate in control cells. However, all the cell types consumed the highest amount of glucose on the first day as illustrated in Fig. 5. It was expected to have higher glucose consumption rate in tamoxifen resistant cells due to the morphological changes and the cells duplication time seen during the repeated experiments.

## Discussion

This study was designed to determine the effect of different tamoxifen resistance development approaches in MCF-7 cell lines by monitoring the molecular changes of *PI3K/AKT/PTEN* signaling pathway and genes related to glucose glutamine metabolism through the treatment process. Interestingly, early molecular changes in *PI3K/AKT/PTEN* signaling pathway preceded resistance development; thus, it would provide potential insight on resistance biomarkers and possible targets to reverse or slow resistance development.

Activation of the *PI3K/AKT/PTEN* signaling pathway has been reported to increase cancer cell proliferation and cellular invasion^16^. *PTEN* encodes a tumor suppressor gene that acts as a negative regulator to PI3K^17^. Its inactivation has been reported in tamoxifen-resistant breast cancer cells^18,19^. Also, *PIK3CA* is a crucial element of this signaling pathway, and it is involved in cell growth, survival, and proliferation. Mutations in this pathway result in improved PI3K signaling associated with cancer^20,21^, leading to endocrine resistance^22,23^. Moreover, changes in *PDK1* and *AKT* gene expression has been reported in tamoxifen-resistant breast cancer^24,25^.

Modulation of gene expression in *the PI3K/AKT/PTEN* signaling pathway before achieving tamoxifen resistance was accompanied by *GSK-3ß* gene expression's downregulation. *GSK-3ß* gene encodes glycogen synthase kinase*.* Once the protein is inactivated by *AKT1 *or the gene is downregulated, glycogen synthase would be converted into its active form leading to stimulation of glycogen synthesis and glucose metabolism^26^. On the other hand, The inactivation of *GSK-3ß* would have been affected the significant increase in glucose consumption rate from culture media that was seen in our study and confirms the results reported by Sokolosky et al. in MCF-7 cell line where an increase in hormonal resistance and decrease sensitivity to targeted therapy was linked to increases glucose consumption^27^.

Another important finding in our study was the increase in *GLUL* gene expression. This increase indicates a higher synthesis rate of glutamine in the cells, and it was confirmed by preliminary data where decreased glutamine consumption rate from culture media was observed, and an increase in glutamine concentration in the media collected from resistant cells were detected, using HPLC/MS–MS, when compared to fresh media and media collected from sensitive MCF-7 cells. It has been reported that luminal breast cancer cells can synthesize glutamine and resist the glutamine less environment through *GLUL* expression^14^, Wang et al. also found that higher expression of *GLUL* in the breast cancer patients was correlated with larger tumor size^28^. However, there were no reports of *GLUL* expression or glutamine consumption in relation to endocrine resistance. *GLUL* over expression was in concordance with *c-MYC* gene expression and would indicate more glutamine synthesis and less uptake from the culture media. *c-MYC* is known to be over expressed in breast cancer, and its involvement in glutamine uptake and degradation is well reported, as it stimulates surface transporters and glutamine synthetase suppression^29^. Chen et al. reported that MCF-7 tamoxifen resistant model produced by treating the cells with increased concentrations of 4-hydroxytamoxifen for 8 months expressed more *c-MYC* and enhanced resistance^30^. Upon comparison with the models produced in this study, much less concentrations of tamoxifen were used with no significant *c-MYC* gene expression and over expressed *GLUL* was observed.

In conclusion, the possible correlation between increased glucose consumption from the media, and increased glutamine synthesis in the cells during the resistance development could be further investigated to determine the potential role of these early metabolic changes in endocrine therapy resistance that could be used as metabolic markers or targeted in the future to re-sensitize or treat endocrine-resistant breast cancer.

## Materials and methods

### Cell culturing and TAM-R development

MCF-7 cell line was purchased from ATCC and was cultured following the standard protocols in RPMI 1640 (EuroClone S.p.A., Italy) media supplemented with 1% penicillin–streptomycin (EuroClone S.p.A., Italy), 1% L-Glutamine (EuroClone S.p.A., Italy), and 10% fetal bovine serum (FBS) (EuroClone S.p.A., Italy). Cells were incubated in a 37 °C incubator under 5% CO_2_ atmosphere.

Two methods of TAM-R development were used in this study. Cells were cultured in two 75-flasks. When the MCF-7 cells were 75–85% confluent, they were treated with gradual doses of Tamoxifen (Santa Cruz Biotechnology, US) starting with a small dose (100 nM) in the first approach and increase the concentrations gradually, as described in the literature^31, 32^. In the second approach, cells were treated with gradual TAM doses, then fixed doses of 35 µM were given several times^33^. Tamoxifen resistant models were produced at three different times and all experiments were repeated in triplicates.

### RNA extraction, cDNA synthesis, and gene expression analysis

RNA extraction from MCF-7 cell lines treated with different TAM concentrations in both resistance development methods was completed using the innuPREP DNA/RNA Mini Kit (Analytik Jena, Germany) following the kit's protocol. After quantification, cDNA was produced using High-Capacity cDNA Reverse Transcription Kit (Thermo Fisher Scientific, USA) according to the manufacturer's procedure. Gene expression analysis was performed with optimized annealing temperatures of primer sets (Supplementary Information) using qRT-PCR CFX96 real-time PCR machine (Bio-rad, USA).

### Flow cytometry

Tamoxifen resistant cells were analyzed using ab14085 Annexin V-FITC Apoptosis Detection Kit (Abcam, UK). Briefly, control MCF-7 cells and TAM-R resistant cells developed by the two approaches were treated with 35 µM tamoxifen and incubated for 24 h in a humidified 5% CO_2_ incubator at 37 °C. Live and dead cells from each sample were then collected by centrifugation, processed as the kit's protocol. Finally, quantification was recorded by BD AccuriTM C6 Plus Flow Cytometry (BD Medical device company, US).

### Glucose metabolism analysis

Glucose consumption from the culture media was detected using Accu-check Performa^34^. The device was standardized using standard glucose concentrations obtained from The Arab Company for Drug Industries and Medical Appliances (ACDIMA), Amman, Jordan, and a calibration curve was used as quality control (QC) (Supplementary Information) in order to use it to detect glucose concentration in media collected from cultured flasks.

One million cells of TAM-R MCF-7 and control MCF-7 were seeded in T-75 flasks then the cells were incubated into CO2 incubator for 3 h. After that, 2 µL of the culture media from each flask were taken every day for four days, and the glucose concentrations were measured using Accu-check Performa. The measurements were repeated as triplicates.

### Statistical analysis

All statistical analysis results were performed using GraphPad Prism 8 via one-way ANOVA or two-way ANOVA. The results were presented as mean ± SD. Statistical significance was represented as *P < 0.05, **P < 0.01, and ***P < 0.001.

## Supplementary Information

Supplementary Information.

## Data Availability

All the authors declare that all data from the study is reported in this article.
