# Supplementary material for: Upregulation of PI3K/AKT/PTEN pathway is correlated with glucose and glutamine metabolic dysfunction during tamoxifen resistance development in MCF-7 cells
Source: Sci Rep. 2020 Dec 14;10:21933. doi: 10.1038/s41598-020-78833-x (PMC7736849; doi:10.1038/s41598-020-78833-x)
Supplement: Supplementary file 1 — Supplementary Information. [file 41598_2020_78833_MOESM1_ESM.docx]

**Upregulation of PI3K/AKT/PTEN Pathway is correlated with Glucose and Glutamine Metabolic dysfunction during Tamoxifen Resistance Development in MCF-7 Cells.**

Lama Hamadneh^1, *^, Rama Abuarqoub^1^, Ala Alhusban^1^, Mohamad Bahader^1^

1. Faculty of Pharmacy, AL-Zaytoonah University of Jordan, Amman, 11733, Jordan

*Corresponding author

[lama.hamadneh@zuj.edu.jo](mailto:lama.hamadneh@zuj.edu.jo)

[ORCID ID: 0000-0003-4672-3303](https://orcid.org/0000-0003-4672-3303)

Supplementary Material

Table 1: Primers sequences of genes used in the study.

| Gene | Forward sequence (5’–3’) | Reverse sequence (5’–3’) | Annealing temperature (°C) | Length of PCR Product |
| --- | --- | --- | --- | --- |
| PIK3CA | CCAGGGAAATTCTGGGCTCC | TGGAATAAGAACTATTCCTGCTCA | 52 | 81 |
| PTEN | ACCAGGACCAGAGGAAACCT | GCTAGCCTCTGGATTTGACG | 60 | 241 |
| PDK1 | GGCGGAAACCCTTGCCTAAC | TATCGCTGCCTCCAAACCTC | 50 | 100 |
| AKT1 | GGAGGTTTTTGGGCTTGCG | CTCTGATGCACCAGCTGACA | 50 | 284 |
| AKT3 | CACCGTCCTCACCCATCAAA | GCCTGGAGTTTGAAATGCACC | 50.7 | 98 |
| GSK3B | CGAGACACACCTGCACTCTT | TTAGCATCTGACGCTGCTGT | 52 | 143 |
| GLUL | GCCTCCTCTCCTCTCCTAACCT | TCATGGTGGAAGGTGTTCTGG | 50.7 | 84 |

**Figure S1** Standard Curve of different serial dilutions from RPMI 1640 media.
